# Crosstalk between adipose tissue and the microbiota-gut-brain axis in metabolic diseases

**DOI:** 10.7150/ijbs.68786

**Published:** 2022-02-07

**Authors:** Ziwei Yu, Yuting Wang, Zhi Yu, Mengjiang Lu, Bin Xu

**Affiliations:** 1 Key Laboratory of Acupuncture and Medicine Research of Ministry of Education, Nanjing University of Chinese Medicine, Nanjing, China.; 2 Nanjing Hospital of Chinese Medicine Affiliated to Nanjing University of Chinese Medicine, Nanjing, China.

**Keywords:** Adipose tissue, microbiota-gut-brain axis, metabolic disease, crosstalk

## Abstract

Recently, the microbiota-gut-brain axis (MGBA) has emerged as a target for therapeutic innovation. Impairment of dynamic relationships within the MGBA promotes the pathological features of metabolic diseases. However, experimental data on the MGBA has limited clinical application. This review summarizes recent studies and proposes that exploring the interaction among peripheral organs and the MGBA could verify the dominant role of the latter in the onset of metabolic diseases and promote the clinical application of research outcomes. We first emphasize the molecular basis of metabolic diseases caused by MGBA disorders, which manifests as bidirectional relationship. We also summarize related therapeutic strategies, along with limitations in their clinical application. Adipose tissue (AT) is dynamic during metabolic activities and might interact with components in the MGBA. Therefore, it is interesting to explore the interplay among the MGBA and different kinds of AT, including thermogenic adipose tissue and white adipose tissue (WAT). In addition, we also evaluate the functional specificity of adipose tissue derived mesenchymal stem cells (ADSCs) and the beige adipose tissue. Understanding the heterogeneity and molecular basis of the interaction between different kinds of AT and the MGBA could accelerate innovation in the diagnosis and therapy of metabolic diseases.

## Introduction

The microbiota-gut-brain axis (MGBA) has consistently attracted attention because of its potential to provide innovative therapeutic strategies for metabolic and neurological diseases, such as obesity, type 2 diabetes mellitus (T2DM), depression, and Parkinson's disease 1-3. There is a dynamic and complex bidirectional network in the MGBA. On the one hand, intestinal microbiota colonized in the gut promotes signaling pathways from the gut to the brain. Not only could the intestinal microbiota participate in digestion and absorption, but directly or indirectly interact with gut epithelium cells by regulating the abundance of metabolites, such as fatty acids, neurotransmitters and secondary bile acids, which maintain the peripheral metabolic status, as well as moving through the gut-brain axis to modulate the central nervous system (CNS) 4-6. The molecular mechanisms of the later interaction have been revealed in recent years, which include immune response, neuronal innervation, and endocrine pathways 5. On the other hand, owing to the blood brain barrier (BBB) and the complex neuron innervation loop inside the brain, the identities and molecular mechanisms of pathways from the brain to the gut within the MGBA remain mostly unknown. In recent years, the development of imageology has revealed that brain-derived signals could regulate gut motility and the composition of the intestinal microbiota through the autonomic nervous system (ANS) 7, which not only constitute bidirectional regulation in the MGBA, but also provides innovative therapeutic strategies for various metabolic diseases, especially obesity.

Adipose tissue (AT) performs essential functions in energy storage and expenditure, as well as metabolic regulation 8. Moreover, AT is also an important endocrine organ. It secretes various cytokines or microRNAs (miRNAs) to interact with distant organs, including the liver, pancreas, intestine, and brain^9^-^12^, which provides a theoretical basis for the interplay among AT and the components or whole structure within the MGBA. In the context of pathology, crosstalk disorders often account for the pathogenesis of obesity and many other metabolic diseases. Previous evidence demonstrates that white and brown adipocytes originate from different progenitor cells, being embodied in the heterogeneity of morphological structures and physiological functions 13. In addition, induced by cold stress or β3-signaling, beige adipose tissue, which are immersed in WAT depots, performs functions in adaptive thermogenesis and energy expenditure, just like BAT 14. However, owing to the differences in celluar origin and thermogenic gene pedigree between beige adipose tissue and BAT, the interaction between beige adipose tissue and surrounding organs may exhibit specificity 13,15-17. Notably, WAT-derived adipose tissue derived mesenchymal stem cells (ADSCs) are active in metabolic processes 18. Their dynamic crosstalk with the MGBA is also a focus of this article. Based on recent research, we first review the crucial role of the MGBA in the development of metabolic diseases, which primarily involve bidirectional signal pathways. Then, we highlight related therapeutic strategies and limitations in clinical applications to emphasize the importance of exploring the external factors that maintain the homeostasis of the MGBA. Finally, we analyze the inter-organizational communication mechanisms between different kinds of AT and the MGBA. Despite lacking adequate literature for the last point, we predict that revealing the mechanisms of the crosstalk between AT and the MGBA could lead to the development of efficient therapies targeting metabolic diseases.

## The MGBA in metabolic diseases

**Figure 1:**
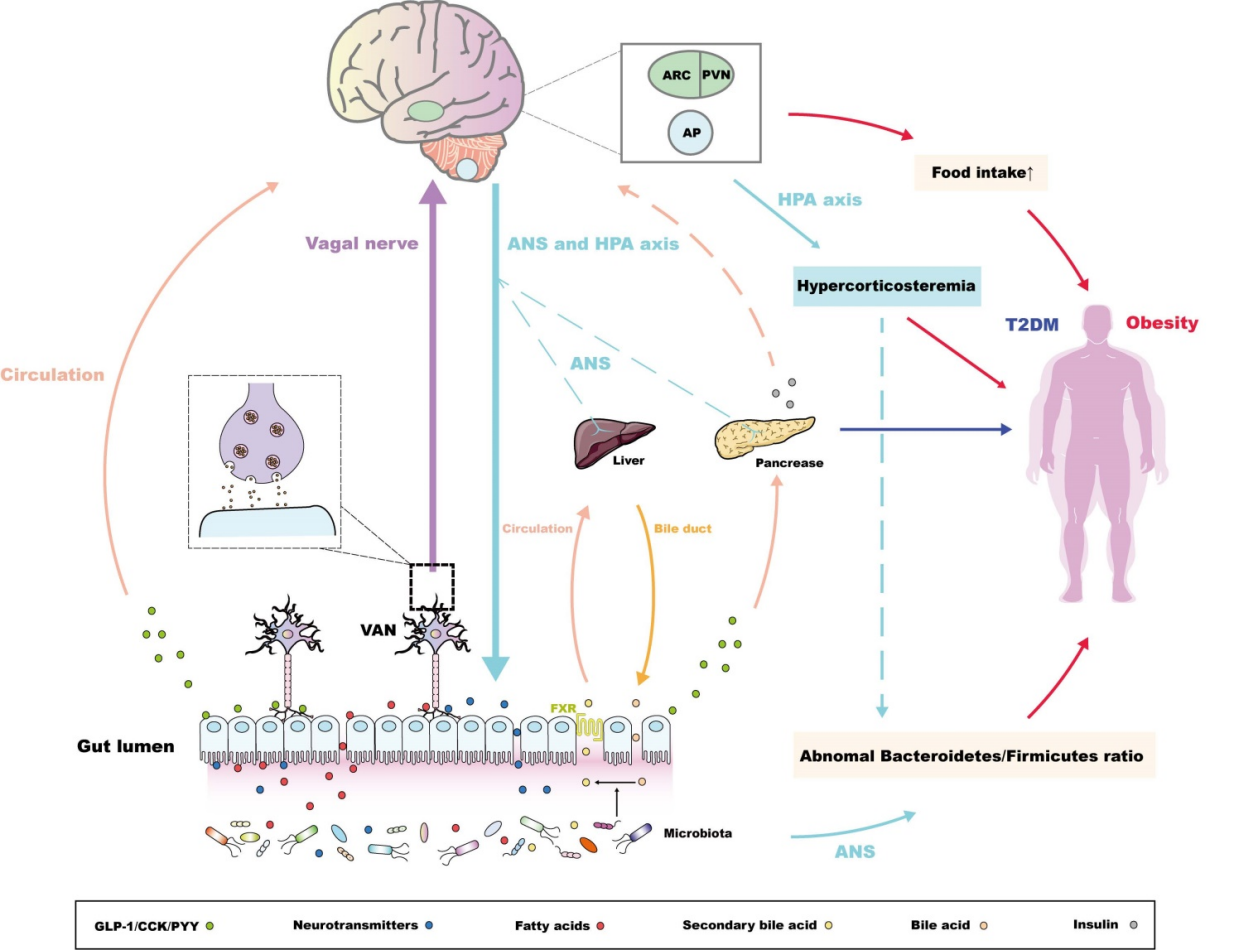
Bidirectional pathways within the MGBA concerning the pathogenesis of metabolic diseases. For the pathway from the gut to the brain, the intestinal microbiota secretes metabolites to promote the release of intestinal peptide hormones and neurotransmitters 37,38,47, along with the conversion of bile acids. The conjugated bile acids are transferred from bile duct and combine the farnesoid X receptor (FXR) in ileal enterocytes to be reabsorbed into the liver via circulation 52. These endocrine signals induced by microbiota could directly or indirectly maintain the peripheral metabolic homeostasis. Alternatively, intestinal microbiota-derived endocrine signals are associated with metabolic-related encephalic regions through the "enteroendocrine cells-enteric nervous system-vagus nerve" pathway 55,56. The molecular pathways via the arcuate nucleus (ARC), the paraventricular nucleus (PVN), and the area postrema (AP) mainly control the balance of appetite, regulating the occurrence and development of obesity 27,35,36,57,58, while these pathways could also regulate glucose uptake of the pancreas, liver through the autonomic nerve system (ANS) 64,65. Notably, the central insulin resistance, which has also been proven to be pathogeny of the T2DM 66,67, is also induced by the abnormal sensitivity or secretion of peripheral insulin initiated by intestinal microbiota or its metabolites 69. For the pathway from the brain to the gut, the anomalous ANS or HPA axis-derived signals could regulate the Bacteroidetes/Firmicutes ratio, which is vital in the development of obesity 77,78. Interestingly, abnormal HPA axis signatures also contribute to hypercorticosteremia, which constitutes risk factors of obesity 78. Following studies could further investigate the relationship between hypercorticosteremia and the 'brain-microbiota-obesity' axis.

The discovery of bidirectional interactions within the MGBA can be traced back to last century. Since the 1980s, it has been reported that intestinal peptide hormones, such as peptide YY (PYY) and cholecystokinin (CCK), constitute the foundation of the gut-brain interaction 19,20, promoting satiety after food intake and the peripheral regulation of CNS functions. Although mainly synthesized and secreted from the gastrointestinal tract 21, these hormones are also influenced by the intestinal microbiota, including *Escherichia coli* 22, or metabolites, exampled by butyrate 23. Studies demonstrated that germ free (GF) animals develop a functional disorder of endocrine peptide hormones, such as PYY 24, and are more susceptible to neurological and metabolic diseases 25-27, which suggest that the gut microorganism ecosystem affects the release of endocrine peptides and maintains the homeostasis of the internal environment, as well as the MGBA. This evidence enriches the connection among the MGBA and metabolic diseases, which has been confirmed in many studies 5,28,29. It also implies that the intestinal microbiota is an initiating factor for gut to brain pathways. Notably, it is estimated that there are more than 10^13^ bacteria in the human colon 30, which contain many more genes than the human genome 31. Not all functions of gut bacteria have been determined; therefore, selecting the key group of intestinal microorganisms for the treatment of metabolic diseases remains a challenge. Evidence also shows that brain-derived signals could regulate the physiological function of the gut 7; however, few studies have summarized the significance of this pathway systematically in the pathogenesis of metabolic diseases. In brief, although recent research has not completely revealed the relationship among the MGBA and metabolic diseases, the MGBA is recognized for its potential to maintain metabolic balance, in which the corresponding pathways mainly include the following two types (Figure 1).

## Gut to brain pathways initiated by intestinal microbiota

As one of the components that first encounter nutrients, the intestinal microbiota not only participates in digestion and absorption, but also acts as an observer of metabolic information 32,33. The latter includes monitoring the glycolipid level and integrating it with the metabolic status of the body, which could be transformed into endocrine or neural signals, eventually modulating peripheral organs 34. In addition, these signals could be transferred to metabolism-related encephalic regions through the MGBA, the molecular mechanisms of which have been summarized in some elegant reviews 5,27,35,36.

### Endocrine signals and peripheral metabolic status

The metabolites released by the intestinal microbiota could influence the physiological feature of orexigenic or anorexigenic peptides, including gastrointestinal tract-derived ghrelin, glucagon like peptide-1 (GLP-1), PYY, and CCK, mainly by controlling the quantity of secretion and adjusting the chemical state of cytokine receptors^37^,^38^. For instance, propionate can induce GLP-1 release via its G protein-coupled receptor and free fatty acid receptor 2 (FFA2) 38,39. These hormones are mainly secreted by enteroendocrine cells, including L cells and K cells, to maintain peripheral metabolic status 5. GLP1 and PYY are reported to be essential in regulating insulin release from islet β cells 40, and a lack of GLP1 in T2DM could lead to the deterioration of glucose homeostasis 41. In addition, CKK also improve metabolic ability by effectively modulating the release of trypsin 42. Compared to control group, glucose is a more potent stimulator of intestinal GLP-1, CCK and PYY after Roux-en-Y gastric bypass (RYGB), which may correspond with higher metabolic requirement 43. Apart from pancreas, recent experiment has replenished the heart and kidney as potential target organs for secretin that has been evaluated to improve cardiac metabolism and renal function 44.

Notably, the intestinal microbiota can interact with neurotransmitters in the intestinal cavity. These neurotransmitters are reported to be dynamic in promoting gut-brain interaction 45. Compared with the control group, there exists significant changes in intestinal neurotransmitters of germ free (GF) mice, including catecholamine, serotonin (5-hydroxytrypta-mine [5-HT]) and gamma-aminobutyric acid (GABA) 46. Fecal purification technique confirms that single-stranded RNA (ssRNA) released from intestinal microbiota could stimulate intestinal epithelial cells to secrete 5-HT through the cation channel Piezo1, which reveals that microbiota is at least partially involved in the production of intestinal neurotransmitters 47. Indeed, the fecal microbiota transplantation (FMT) from lean donors could improve insulin sensitivity in patients with metabolic syndrome, which is closely related to GABA from microbiota, especially *Lactobacillus brevis* 48. Norepinephrine and 5-HT modulate bacterial motility and virulence via quorum sensing mechanism to affect the metabolic state of peripheral organs 49,50. In addition, microbiota-induced 5-HT synthesis is also vital in peripheral glucose handling after antibiotic treatment 51.

In addition, the bile acid signature determined by intestinal microbiota also contributes to the homeostasis of peripheral metabolic status. During food intake, conjugated bile acids from the liver enter the intestine through the bile duct, assisting the digestion of lipid food, and are converted into secondary bile acids, including deoxycholic acid and lithocholic acid, through the modulation of microbiota. Subsequently, these secondary bile acids are effectively reabsorbed (> 95%) into the liver in the ileum and colon through activating the farnesoid X receptor (FXR) 52. The key intestinal microbiota that mediates bile acids has not been identified so far. However, microbial structural variants (SVs), highly variable segments released from microbiota, have been proven to play a key role in human BA metabolism 53. Variation of colic acids proportion and colic 4 level in plasma mediated by an SV of B. *wexlerae* reveal the mechanism of metabolic disorders caused by excessive intake of red wine and soda 53. Similarly, in RYGB or sleeve gastrectomy (SG), the weight loss effect is also related to changes of intestinal bile acid pool, which are attributed to variation of the *Enterobacteriaceae* family 54. In conclusion, intestinal hormones, neurotransmitters and bile acids induced by microbiota constitute dynamic functional units of the MGBA and play key roles in maintaining peripheral metabolic homeostasis.

### Neural signals and central metabolic integration

In metabolic diseases, the disorder of endocrine signals not only disturbs the peripheral metabolic state, but also promotes the transmission of neural signals through binding the surface receptors of enteric neuron or vagal afferent neuron (VAN). For instance, the neuroanatomy of adult enteric nervous system (ENS) is determined by the release of gut 5-HT and the activation of 5-HT_4_ receptor 55. What's more, ghrelin regulates normal feeding and metabolic function by combining its receptor (GHSR) expressed on VAN 56. As for neural signals, obesity-induced abnormal endocrine signature can act on encephalic regions, such as the arcuate nucleus (ARC) and paraventricular nucleus (PVN) in the hypothalamus or the area postrema (AP) located on the caudal brainstem, through circulation or the “enteroendocrine cells-enteric nervous system-vagus nerve” axis. This eventually damages the appetite balance and satiation monitoring within the CNS, or affects the limbic system of the midbrain, which reduces sensitivity to high-energy food, promoting the development of glycolipid metabolic disorders 27,35,36,57,58. In addition, the intestinal hormone secretin, as well as the microbiota metabolite butyrate, improve the thermogenesis of brown adipose tissue (BAT) by regulating the ANS output to maintain the metabolic balance 59,60, which consolidates the dominant place of the MGBA in metabolic diseases. This pathway will be comprehensively explained later in this article. Indeed, the turnover rate of central dopamine and 5-HT also change in GF mice, which leads to dopamine-mediated central dietary reward behaviour or 5-HT-induced autism and its related metabolic complications 61,62.

Concerning diabetes, on the one hand, the decline of butyrate biosynthesis and the deterioration of oxidative stress originating from the damage of intestinal environment homeostasis accounts for the proinflammatory state characterizing diabetes 32. On the other hand, the intestinal microbiota, exampled by the phylum *Proteobacteria* (*Bradyrhizobiaceae*, *Bosea*, and *Sphingomonas*), monitors the sugar content in food and circulation simultaneously, and adjust its compartmentalization in response to glucose fluctuation 40,63. In addition, the intestinal microbiota, especially *lactobacilli* (*L farciminis, plantarum* and *fermentum*), induces endocrine signals, such as nitric oxide, at the ENS through the same pathways as those in obesity to regulate glucose uptake in the liver, pancreas, and adipose tissue by adjusting the ANS output 64,65. In addition, central insulin resistance is one of the new pathogeneses of T2DM, which is caused by the decrease of central insulin sensitivity 66,67. The insulin in the CNS not only originates from the central synthesis of insulin, but also from circulating insulin penetrating through the BBB 64,67,68. The role of the intestinal microbiota in the latter pathway has not been proved directly; however, evidence shows that altered intestinal microbiota and consequently altered metabolites, including short-chain fatty acids (SCFAs) and amino acid, are associated with peripheral insulin sensitivity and circulating insulin levels in patients with obesity or T2DM 69, which emphasizes the potential of the intestinal microbiota to induce central insulin resistance. This could reveal the connection between the MGBA and T2DM, which is initiated by the intestinal microbiota.

## Brain to gut pathways induced by brain-derived signals

The brain to gut pathways induced by brain-derived signals also contribute to dynamic functions of the MGBA. Early brain imaging studies showed that CNS signal variation, deriving from human emotional and cognitive changes, can be transmitted through autonomic nerves to regulate food intake and gastrointestinal contractile function 7, the mechanisms of which include regulating sympathetic and parasympathetic tone in the ANS later 70,71. These mechanisms mainly reveal the pathogenesis of many functional gastrointestinal diseases (FGIDs), including irritable bowel syndrome (IBS) and functional dyspepsia 72-74; however, just few studies have directly determined influences of the brain to gut pathways in the pathogenesis of metabolic diseases. Notably, the FGIDs induced by chronic stress are often complicated by appetite disorders and anomalous glucose and lipid metabolism 75,76, which eventually promote concomitant metabolic diseases. Hence, the brain to gut pathways within the MGBA constitute indirect factors that promote the development of metabolic diseases.

In addition, compared with the control group, GF mice transplanted with an 'obese microbiota' displayed an obvious increase in whole body fat, which could be attributed to the abnormal *Bacteroidetes*/*Firmicutes* ratio in the gut 77,78. Significantly, brain-derived signals could also be regulators of the intestinal microbiota. For instance, traumatic brain injury (TBI) reduces the diversity of fecal microbiota, and the degree of decrease is positively correlated with the severity of TBI 79. In addition, some studies have confirmed that chronic stress, including major depressive disorder (MDD), could change the compounds related to the metabolism of tryptophan and bile acids, and consequently alter the intestinal microbiota at the level of phylum and genus, which might be related not only to dysfunction of the HPA axis, but also to impaired autonomic nerve signals, as well as immune system disorders 80,81. Obesity is closely related to changes of the intestinal microbiota; therefore, we speculated that brain-derived signals within the MGBA could also result indirectly in metabolic diseases by adjusting the diversity or metabolites of the intestinal microbiota. Unfortunately, the exact mechanisms of this 'brain-microbiota-obesity' axis have not been determined. In conclusion, studies have gradually confirmed that brain-derived signal stabilization is also one of the important factors in maintaining MGBA homeostasis. This route is often associated indirectly with the incidence of metabolic diseases, especially obesity. Future work should focus on the anomalous signals derived from the HPA axis in chronic stress, which are not only core regulators of the intestinal microbiota, but also the chief producers of the hypercorticosteremia that causes obesity 78. Furthermore, it is meaningful to illustrate the relationship between hypercorticosteremia and the 'brain-microbiota-obesity' axis (Figure 1).

## Current therapeutic strategies and their limitations

### Lifestyle improvement and fecal microbiota transplantation based on gut to brain pathways

The molecular mechanisms of the gut to brain pathways within the MGBA provide scope for improvement of therapeutic strategies against metabolic diseases. First, studies based directly on the intestinal microbiota have illustrated that bacterial diversity, along with its metabolites, are closely related to diet and exercise 82,83. The dominant position of diet has been proven by many related studies. For example, food originated polyphenols increase the number of beneficial species, such as *Bifidobacterium*, *Lactobacillus*, and *Faecalibacterium prausnitzii*, which suggests a good application prospect in the treatment of metabolic diseases 84,85. In addition, tributyrin, as a food additive, has been proven to restore the immune function of the intestinal microbiota in obesity, which is an important research target for the development of new therapeutic strategies for overweight people 86. However, differing from a previous conclusion, an experiment using metabolic profiling combined with a mathematical modeling strategy demonstrated that although a healthy diet alone can promote the optimal release of bacterial metabolites, represented by SCFAs, tryptophan and phenylalanine, the combined effects of exercise and dietary changes, rather than just healthy eating, increased intestinal microbial diversity and promoted the expression of medium-chain fatty acids (MCFAs) and branched-chain SCFAs 87. This provides reliable design methods and general thoughts for future research targeting intestinal bacteria. Nevertheless, the increasing bacteria diversity is not proportional to the expression of healthy metabolites, because intestinal protein metabolism is often related to the formation of toxic compounds 88. Thus, it would be worth exploring the maximization of the relationship between exercise, dietary, and metabolic diseases on the basis of existing control variable techniques.

Secondly, compared with the diet and exercise, fecal microbiota transplantation (FMT), has become a hot research topic in recent years, the effectiveness of which in the treatment of metabolic diseases, including obesity and irritable bowel diseases, such as ulcerative colitis and IBS, has been proven in some randomized controlled trials 89-91. However, in a small pilot study of canine acute hemorrhagic diarrhea syndrome, FMT only resulted in an increase in microbiota diversity, but had no clinical benefits 92, which might be attributed to a lack of comprehensive screening criteria for donors. Hence, this technique requires standardization in experimental design 93, because the host aberrant information carried by the fecal microbiota might impair the homeostasis of the internal environment within the receivers, for example constipation-induced gut microbiota dysbiosis aggravates the regulatory T cell (Treg)/T helper cell 17 (Th17) and Treg17/effector Th 17 (Teff17) imbalance and disturbs cytokines, eventually exacerbating encephalomyelitis (EAE) 94. Future work should concentrate on selecting a donor with a normal dysbiosis index and a favorable specific microbial signature, along with precisely defining the optimum treatment intensity of microbiota transplantation 89,90. In addition, because of the cautious attitude of clinicians regarding the safety of FMT, as well as the lack of continuous follow-up studies to validate the consequences of changes in human microbiota, the clinical indications of FMT are only recommended for the treatment of recurrent *Clostridioides difficile* infection 95. Recently, an interesting experiment highlighted that FMT can exert its therapeutic potential mainly by reversing the relevant miRNA (especially miR-22-3p and miR-451a) changes in the disease state, which provides more powerful mechanistic support for the clinical application of FMT 96. In addition, FMT acts not only on the intestinal environment, but also on the MGBA to regulate the CNS, the indications of which involve EAE, depression, anxiety, and autism spectrum disorder 94,97,98. Future work should pay attention to the potential impact of FMT on the modulation of metabolism-related encephalic regions to expanding the utilization of FMT in metabolic diseases. Researchers can take inspiration from the fact that FMT has a wide range of indications in animal experiments. Hence, translating basic research achievements into clinical applications is the most important problem to be solved.

## Imaging technology and neural recording instruments based on brain to gut pathways induced by brain-derived signals

Emotional factors contribute to adverse symptom severity and health-related quality of life for patients with metabolic diseases; therefore, the utilization of antidepressants and psychological therapies has become more popular in recent years 99,100. However, these therapeutic strategies can only relieve emotion-related complications of patients, there is little evidence supporting their use to treat active disease, or to maintain disease remission 74, which reveals that the key routes concerning brain-to-gut signals that induce metabolic diseases have not been determined. This could probably be attributed to whether CNS signal changes are the cause, consequence, or incidental to the metabolic diseases in question, which remains largely uncertain. Moreover, the complex crosstalk involving the MGBA makes it difficult to establish a specific correspondence between a CNS pathological pathway and metabolic diseases. To fill this blank, brain imaging technology has been constantly employed, and its continuous refinement and innovative design over the decades has increased our understanding of neural signals. Interestingly, in addition to the constant innovation of brain imaging technology, including the application of H(2)(15)O positron emission tomography and functional magnetic resonance imaging 101,102, advances in neuroengineering have also received widespread attention, in which the instruments used to record neural signals, such as frame-projected independent-fiber photometry (FIP) microscopes, are able to project dynamic activity signals from many distant, deep brain areas onto the camera to detect the physiological response of neurons 103. Furthermore, the memristor has been proven to be efficient in synapse simulation and for computing neural networks *in vivo* 104,105. Although the memristor is mainly employed in the field of artificial intelligence, its recording mode of neural signals clearly revealed the specific components of the signal transduction pathway. Therefore, research on the treatment of metabolic diseases from the CNS could be combined with the development of modern technology, applying the signal transduction pathway as the medium to explore the deeper connection within the MGBA and to optimize therapeutic strategies.

In summary, functional disorders of the MGBA, which are reflected in both peripheral and central aspects, are deeply involved in the development of metabolic diseases. Although our increasing knowledge regarding signal pathways provides many potential approaches for the treatment of metabolic diseases, analysis of the above data shows that there is still a long way to go before scientific achievements, including FMT and neuroengineering products, achieve clinical popularity. In addition to safety limitations, the lack of recognition of the crosstalk among the MGBA and other organs should be considered as well, because the pathogenesis of metabolic diseases often involves multidirectional interactions among various peripheral metabolic organs 106. Hence, it is necessary to explore the external factors that maintain the homeostasis of the MGBA to seek more efficient signaling pathways and optimize the therapeutic strategies of metabolic diseases.

## The crosstalk between adipose tissue and the MGBA

Adipose tissue acts as an essential organ in regulating energy and glucose homeostasis, which provides evidence for the subtle connection with metabolic diseases, and this metabolic regulating function has been proven also dynamic in humans 107. From the anatomical point, the abundant neural fibers in AT provide a basis for the direct or indirect crosstalk between AT and the neural circuits of the MGBA 108. In addition, the endocrine interaction between AT and peripheral organs also maximizes the chance of identifying therapeutic molecular targets and strategies for various diseases 9-12. Hence, it is worth exploring the interplay among different kinds of AT and the components or whole-structure of the MGBA. In the following section, we review the role of AT in maintaining the functional homeostasis of the MGBA, with providing novel potential targets for the treatment of metabolic diseases and accelerating the translation of experimental achievements into clinical applications. However, owing to the limited functional conservation of AT between experimental animals and humans, more experiments are required to explore the biological significance of these pathways in the treatment of metabolic diseases.

### Thermogenic adipose tissue

Thermogenic adipose tissue, BAT and beige adipose tissue, is the core organ of non-shivering thermogenesis, the heating microenvironment homeostasis of which protects the body against cold and other extreme environments 109,110. The thermogenic adipose tissue has abundant neural fibers, the efferent branches of which have been proven to possess sympathetic activity, constituting the sympathetic nervous system (SNS). Generally, nutrient transportation to the alimentary canal mediates the secretion of gastrointestinal hormones, including CCK, GLP1 and secretin, which cooperate with metabolites (especially fatty acids, neurotransmitters, and secondary bile acids) induced by the microbiota to optimize systemic metabolism in the whole body 5,111. The process above is closely related to the MGBA and thermogenic adipose tissue-derived SNS, which is determined by the fact that thermogenic adipose tissue, especially BAT, can not only serve as the terminal target of the MGBA, but also as a mediator to consolidate crosstalk between the gut and the brain. In addition, BAT also secretes low levels of efficient adipokines, such as neuregulin 4 (NRG4), bone morphogenetic protein 8b (BMP8B) and calsyntenin 3β, along with inflammatory cytokines, including interleukin (IL)-6 112-114, which allow it to maintain the homeostasis of the MGBA.

### BAT acts as a terminal target of the gut-brain interaction

**Figure 2:**
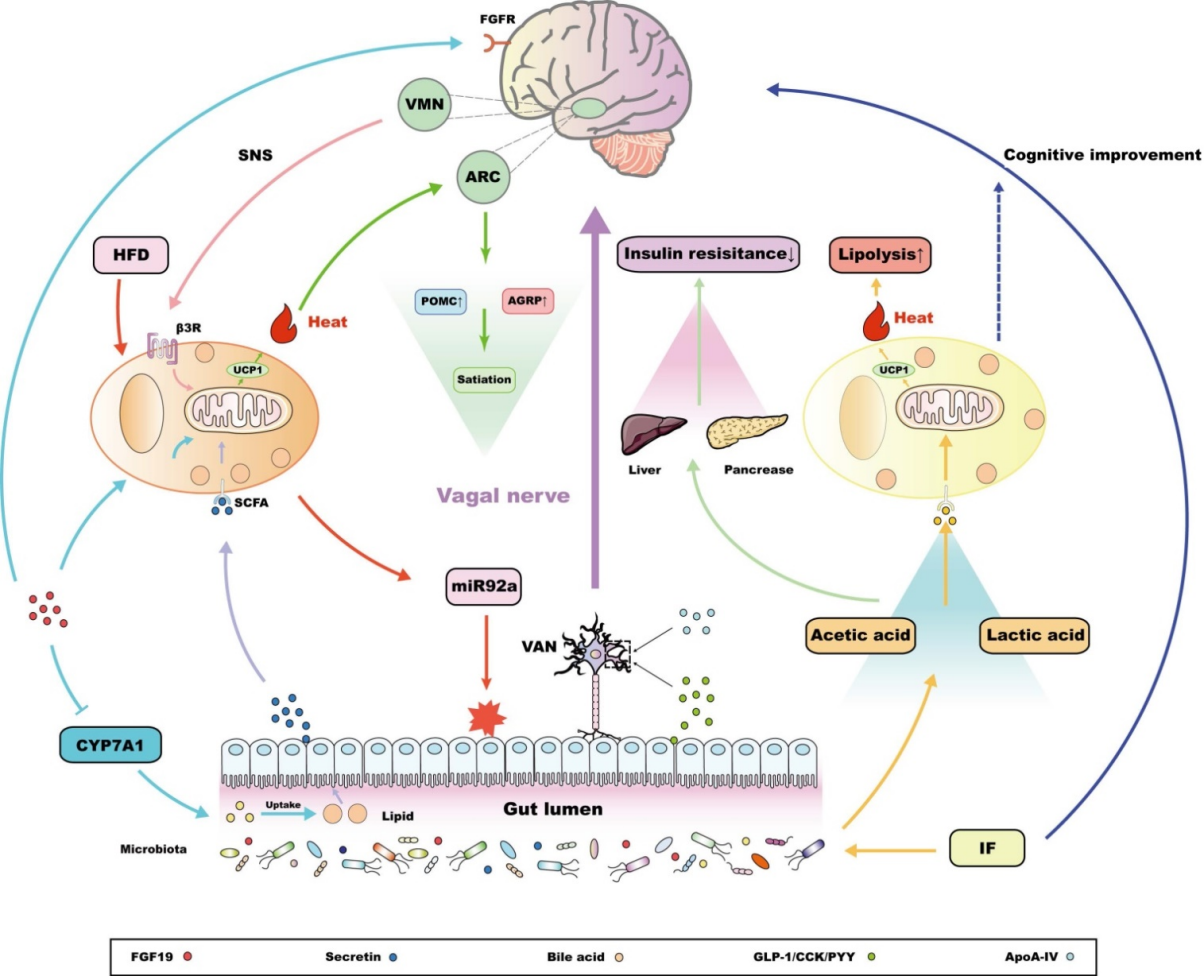
The interaction between thermogenesis adipose tissue and the MGBA. Intestinal hormones, together with circulatory ApoA-IV, could act on related receptors, located in the vagal afferent neuron (VAN), which could connect with the ventromedial hypothalamic nucleus (VMN) and drive the sympathetic nervous system (SNS), finally promoting the adaptive thermogenic function of brown adipose tissue (BAT) 117-120. In addition, ileum-derived FGF19 could promote the metabolic function of BAT in a UCP1-dependent manner 126,127. The metabolic benefits of FGF19 are also shown in the inhibition of the key enzyme CYP7A1 associated with bile acid synthesis in liver, which, in turn, reduces lipid uptake in gut 127. Meanwhile, central FGF receptors also determine the metabolic regulating potential of FGF19 128. Notably, secretin released from the intestine after food-intake enters the circulation and acts on the secretin receptors (SCTRs) in BAT, promoting thermogenesis, the Increasing heat of which could activate central transient receptor potential vanilloid 1 (TRPV1) receptors, eventually inducing satiety 59,130-132. In addition, a high fat diet (HFD) promotes BAT to release miR92a, which damages the intestinal environment and has been reported as a risk factor for adverse outcomes, such as colorectal cancer 137,139,140. Interestingly, intermittent fasting (IF) has been reported to regulate the release of metabolites to promote the browning of white adipose tissue (WAT), and eventually increase lipolysis in the liver and pancreas 146. This process is independent of BAT and could be a functional specialty of the beige adipose tissue. Some studies have found that IF can also improve cognitive function 147, and whether this central optimization is related to the function of beige adipose tissue is worth exploring.

According to the nutrient status or dietary preference, the neurochemical phenotype of the intestinal VAN changes when combined with different kinds of intestinal peptide hormones, submitting orexigenic or anorexigenic signals to the CNS, the feedback of which, in turn, regulates gastrointestinal function 115,116. The lipid variation sensed by the duodenum could promote BAT thermogenesis, which is attributed to the ventromedial hypothalamic nucleus (VMN) activated by vagal neuron cholecystokinin A (CCKA) receptors to modulate the tone of the SNS output 117. In addition, apolipoprotein A-IV (ApoA-IV) knockout mice could reverse this situation, suggesting that CCKA receptors might rely on ApoA-IV to activate thermogenesis in BAT and promote energy consumption 118,119. Similarly, intestine‑derived peptide hormones, such as GLP-1, can also induce thermogenesis of BAT through the signal transduction from the intestinal VAN to the CNS, in which VAN GLP-1 receptor signaling is reported to make a great contribution 120. In fact, the activation of BAT thermogenesis after bariatric surgery is also related to an increase in intestinal GLP-1 production 121. Interestingly, GLP1 receptors are widely expressed in the brain, and central GLP1 injection is sufficient to increase SNS output and activate BAT thermogenesis 122. However, GLP-1 is susceptible enzymatic degradation by dipeptidyl peptidase-4 in the periphery 123; therefore, diet-induced GLP-1 activates BAT thermogenesis through signal transmission of VAN rather than flowing into the blood stream to directly communicate with central GLP-1 receptor. This kind of facultative BAT-associated thermogenesis induced by food intake is collectively referred to as diet-induced thermogenesis (DIT) 124. This establishes the concept of the gut-brain-BAT axis (Figure 2), which not only reveals a self-protective mechanism against obesity induced by food load, but provides an efficient approach to regulating BAT function in addition to cold challenge, demonstrating an innovative therapeutic strategy for metabolic diseases on the basis of the crosstalk between BAT and the MGBA.

In addition, produced by ileal enterocytes, fibroblast growth factor 19 (FGF19) and its mouse orthologue FGF15, are favored in the treatment of metabolic diseases. FGF19/15 are satiety hormones, and are negatively correlated with BMI and fat mass, while positively correlated with the expression of UCP1, which is the marker gene in BAT and beige adipose tissue 125. Exogenous FGF19 is reported that not only promotes insulin stimulated glucose dispose in BAT, but efficiently improves the energy consumption, which is relevant to the up-regulation of UCP1 in BAT 126,127. Interestingly, in UCP1 knockout mice, FGF19 treatment still appears to have anti-obesity action and increase fecal energy content, which is attributed to the inhibition of the key enzyme CYP7A1 associated with bile acid synthesis, thus reducing the lipid uptake in gut 127. Apart from this, systemic FGF19 administration effectively improves glucose tolerance in ob/ob mice, which can be attenuated by inhibition of central FGF receptors 128. This reveals that the metabolic regulation of FGF19 may also be associated with the CNS. The evidence mentioned above implies that the activation of BAT induced by FGF19 may also partially from DIT based on the MGBA, as serum FGF19 level directly affects muscle sympathetic nerve activity (MSNA) in patients with obesity 129. In this work, the correlation between FGF19 and MSNA frequency is reported to be negative 129. However, considering tissue specificity, following studies could further explain the mechanism of the connection between BAT and the MGBA mediated by FGF19 via the neuroendocrine aspect, and how to apply this connection to the treatment of metabolic diseases.

### BAT acts as a mediator for central metabolic signals regulated by intestinal hormones

In 2018, an elegant report revealed that intestinal hormones, especially secretin, released by diet stimulation, promoted thermogenesis in BAT, which could activate transient receptor potential vanilloid 1 (TRPV1) expressed in proopiomelanocortin (POMC) neurons and then promote satiation 59. Although this process could also be classified as DIT, BAT is not a target, but an intermediate organ, the thermogenic function of which ultimately regulates appetite and could be modulated to treat metabolic diseases, especially obesity (Figure 2). Notably, the thermogenic activation of BAT induced by secretin depends on lipolysis-igniting uncoupling protein 1 (UCP1) 59. UCP1 is a downstream effector of the SNS in BAT 130; therefore, it was speculated that secretin-induced DIT requires the integrity of the SNS pathway. Following clinical randomized controlled trials demonstrate that, in human, secretin also ignite metabolic regulation feature of BAT, which not only optimizes glucose uptake efficiency in BAT, but suppresses appetite, as well as delaying resumption to eat after a meal 131. Unlike the mechanism by which GLP-1 and CCK induce thermogenesis in BAT, the non-selective beta blocker propranolol did not affect the thermogenesis in BAT and central satiation variation induced by secretin, ruling out the regulatory role of the gut-brain-BAT axis in terms of BAT thermogenesis induced by secretin 59,132. In this context, BAT could act as a medium for intestinal hormone regulation of central metabolic signals. Similarly, considering that the thermogenesis of BAT will eventually influence central satiation, the gut-brain-BAT axis activated by GLP1 and CCK might eventually target the hypothalamic appetite regulation area, which cooperates with their direct effects on the metabolism-related encephalic regions to simultaneously regulate satiation. However, more experimental studies are required to reveal the mechanistic details of the gut‑brain-BAT-satiation axis. Therefore, BAT not only act as the target organ of gut‑brain communication, but also exerts its own functional feedback to maintain the homeostasis of the MGBA.

### BAT maintains the homeostasis of the MGBA

Adaptive thermogenesis and the promotion of energy expenditure are the main functions of BAT and the secretory profile of BAT has been highlighted in recent years 112. Unfortunately, although the endocrine factors secreted by BAT could maintain SNS innervation in BAT, exampled by S100B, NRG4 and nerve growth factor (NGF) 114,133,134, or carry inter-organ information, such as BAT-secreted NRG4, which targets the liver and inhibits hepatic lipogenesis 135, there is little research on the relationship between BAT secretory factors and the MGBA at the molecular levels. In one example, BAT-derived BMP8B could cross the blood-brain barrier, where its acts on the VMN and the ARC to regulate SNS output 136. Nevertheless, further verification is required to explore whether this kind of central activation is related to the homeostasis of intestinal function and the gut microorganisms ecosystem.

In addition to proteins, BAT also secretes miRNAs, which exist in the exosomes of brown adipocytes and are secreted by BAT under certain environmental stimulations 137. Chen and collaborators proved that the miRNA in the exosomes derived from BAT mainly include miR-92a, miR-133a, and miR-34c*, among which only miR92a is conserved between mice and humans, serving as a biomarker for BAT function. In fact, there was an inverse correlation between miR92a levels and the activity of BAT 137. In addition, the increase in miR92a levels after high fat diet (HFD) promoted the process of 'whitening' of BAT, which was consistent with the decrease of miR92a to a standard level after bariatric surgery 137,138, ultimately improving metabolic status. It is worth noting that miR92a, which targets at ING2 mRNA, has been classified as a carcinogenic factor and is closely related to the occurrence and progression of gastric and colon cancer^139^,^140^. Moreover, overexpression of miR92a reduced the amount of butyric acid secreted by intestinal microorganisms 141, thus interfering with the function of the MGBA in microbiota metabolism, which will lead to adverse outcomes, such as colon cancer (Figure 2). Combined with the above analysis, miR92a interferes with the homeostasis of the MGBA, which could be reversed by BAT activation of BAT, thus indirectly inducing the recovery of related metabolic diseases.

### Functional specificity of beige adipose tissue

Originating from the browning of WAT, beige adipose tissue expresses a low basal level of UCP1, but is somewhat sensitive to cAMP signaling, which is associated with thermogenesis 142. Hence, it is also classified as thermogenic adipose tissue. Although the chromatin landscape covering epigenetics and transcriptional regulation is similar in beige adipose tissue and BAT 143, beige adipose tissue develops from non-dermomyotome lineage after birth, which is essentially different from BAT 17. This difference in celluar origin results in functional heterogeneity between beige adipose tissue and BAT, which is reflected in different adipocyte population and thermogenic gene pedigree 15,16,144,145, suggests that there is specificity in terms of the interaction between beige adipose tissue and the MGBA. Li and colleagues found that every-other-day fasting (EODF) altered the composition of the intestinal microbiota, resulting in increased levels of the fermentation products acetic acid and lactic acid, which promote the development of browning of WAT into beige adipose tissue, and eventually ameliorate obesity, insulin resistance, and hepatic steatosis in a BAT-independent way 146. Interestingly, intermittent fasting (IF), including EODF, has been proven to improve cognitive function, which is associated with the reconstruction of cognitive function-related intestinal microbiota metabolism 147. In this way, an IF induced gut-to-brain axis has been established, the peripheral metabolic benefits of which are relevant to the activation of beige adipose tissue. Although the effect of beige adipose tissue in this process has not been completely determined, there is a possibility that it serves as a medium for IF-induced gut-brain communication, promoting the pathogenesis of metabolic diseases (Figure 2).

### WAT

WAT is one of the largest metabolic organs in the body, and is involved in physiological processes such as energy storage and metabolism 8,148. With a more comprehensive secretory function than BAT 112, WAT might have a more extensive interaction with the MGBA. Hence, we primarily review the general crosstalk between WAT and the MGBA in the following section, which involves a complex bidirectional regulation at the intersection of metabolism and neuroimmunology. Subsequent research on the function of WAT should focus on the pivotal cytokines in this tissue to optimize the treatment of related metabolic diseases.

### The MGBA maintains the lipid storage capacity of WAT

**Figure 3:**
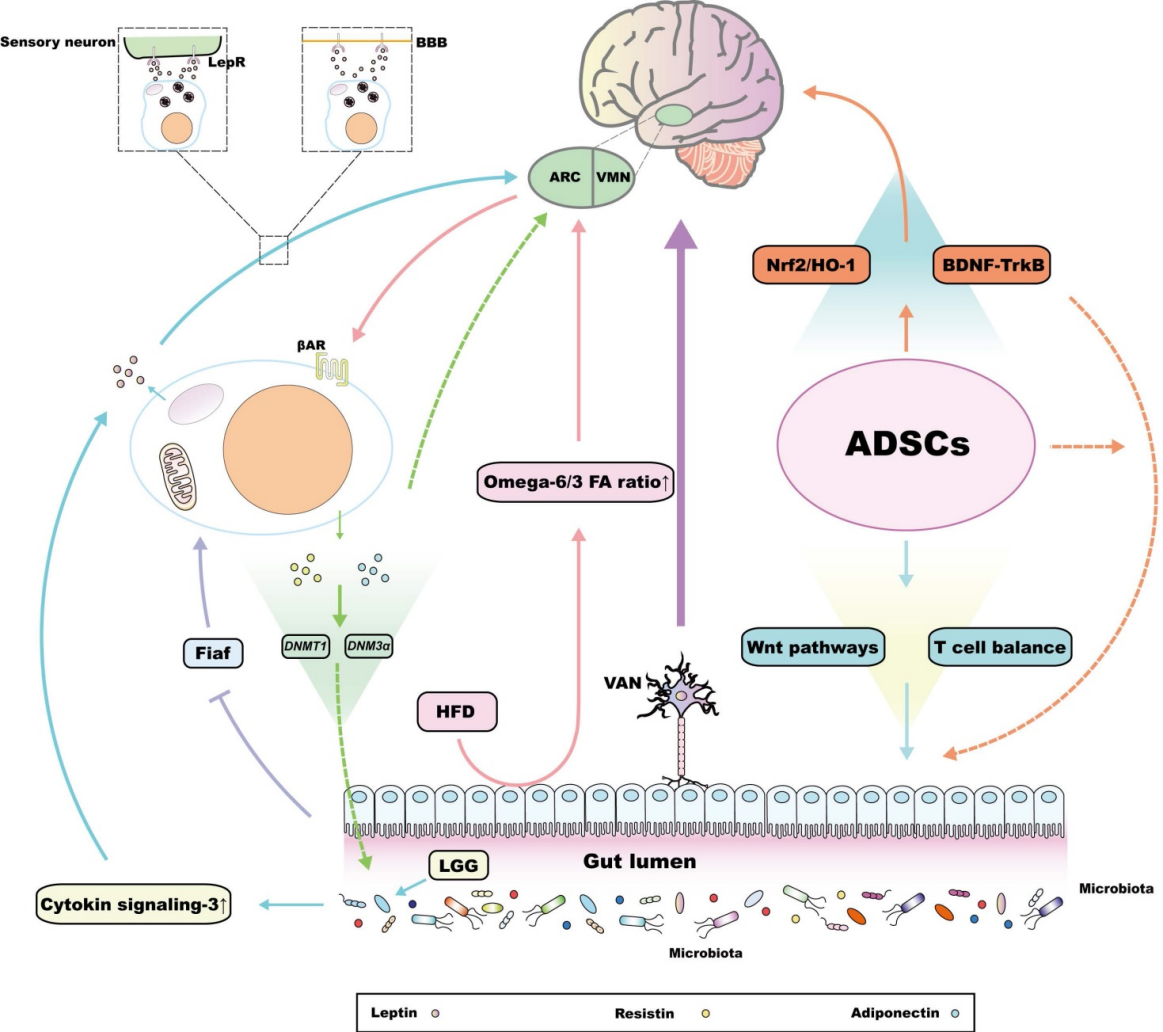
The interaction between white adipose tissue (WAT) and the MGBA. In GF mice, excessive release of fasting-induced adipocyte factor (Fiaf) from the microbiota leads to the rapid production of visceral WAT (vWAT) 149. In HFD mice, the increasing intestinal omega‑6/omega-3 fatty acid (FA) ratio often directly affects the arcuate nucleus (ARC) and the ventromedial hypothalamic nucleus (VMN), promoting fat accumulation in WAT 155,156. Significantly, leptin released by WAT can regulate the output of the autonomic nerve system via acting on the hypothalamus. In addition to directly passing through the blood brain barrier (BBB), peripheral leptin has also been reported to bind to leptin receptors (lepR) on the presynaptic membrane of sensory neurons in WAT to connect with relevant encephalic regions 8,9. Exogenous LGG has also been proven to regulate the composition of intestinal microbiota and WAT‑derived leptin resistance by promoting hypothalamic cytokine signaling-3, which reveals the potential of WAT as a medium to contact with components within the MGBA 166. Interestingly, the other WAT‑derived adipokines, such as adiponectin and resistin also interact with the metabolic homeostasis of the gut and brain, the molecular mechanism of the former is associated with the *DNMT1* and *DNMT3a* genes located around their DNA promoters 169,170. In addition, adipose tissue-derived mesenchymal stem cells (ADSCs) originating from WAT play a unique role in interacting with the MGBA. Not only could they protect intestinal mucosa against inflammatory injuries by rebalancing the T cell repertoire and activating noncanonical Wnt signal pathways 185, but also can regulate inflammation in the central nervous system (CNS) through the brain-derived neurotrophic factor (BDNF)-tropomyosin related kinase B (TrkB) pathway or by upregulating Nrf2/HO-1 signaling 186. Interestingly, these latter two central pathways have also been reported to maintain intestinal metabolic homeostasis 191-193.

The interaction between WAT and the MGBA has a bidirectional form. First, the MGBA maintains the lipid storage capacity of WAT. On the one hand, the intestinal microbiota might affect lipid metabolism, which is involved in adjusting the fat mass and regulating lipid aerobic oxidation. Transplanting a normal microbiota from the distal intestine of conventionally raised mice into GF mice contributed to rapid hepatic lipogenesis and insulin resistance, which is caused by the inhibition of fasting-induced adipocyte factor (Fiaf) in GF mice after bacterial transplantation 149. Fiaf is a circulating lipoprotein lipase inhibitor, and is suppressed in normal animals but overexpressed in GF mice 149, which reveals the mechanism of the metabolic dysfunction in GF mice after microbiota transplantation. Randomized controlled trials also showed that the transfer of stools from patients who have received bariatric surgery, rather than those from obese patients, into GF mice reduced fat deposition and the respiratory quotient 150. As the stool reflects the dynamic changes of the intestinal microbiota of the patient, this experiment also illustrates that the intestinal microbiota determines the lipid metabolism. On the other hand, different kinds of fatty acid (FA), which are involved in daily intake, produce various appetite signals as well as regulating WAT fat accumulation via communication between the gut and the brain. For example, in an HFD, the ratio of omega-6/omega-3 FA is much higher than the 1:1 level in a chow diet, which promotes fat accumulation and increases the risk of obesity 151,152. Mechanistic analysis showed that the FA absorbed through intestinal cells is released into the blood, penetrating the BBB through passive diffusion 153,154, and is finally recognized by various nuclei in the hypothalamus, such as the ARC and the VMN 155,156, via binding transporters such as fatty acid translocase (FAT; also known as CD36), thus modulating food intake and fat decomposition 154. Excessive omega-6 FA not only impairs the sensitivity of the hypothalamic FA detection system, but also affects the inflammatory state downstream of the MGBA, promoting fat mass growth and inflammation in WAT, which ultimately leads to obesity and cardiovascular disease 154,157. In fact, intracerebroventricular injection of unsaturated FA, rather than saturated palmitic acid (PA), produced stronger anaerobic signals in POMC neurons, decreasing weight and fat mass 158. In summary, the components of the MGBA are involved in the regulation of fat reservation in WAT, which not only modulates the central energy intake and lipid growth signals, but also regulates fat mass at the genetic level (Figure 3).

### WAT-derived leptin is the core endocrine factor that promotes the interaction between WAT and the MGBA

Several endocrine factors derived from WAT flow into the bloodstream and reach distant organs 9,10, eventually modulating the physiological functions of the MGBA. Leptin is a classical endocrine factor, which in rodents, is predominantly secreted from visceral white adipocytes, playing a central role in the management of energy storage and the resistance to obesity 9. Adipose-derived leptin has a vital role in regulating central sympathetic outflow, the mechanism of which varies in different adipose tissues 159. According to recent research, two pathways account for adipose tissue‑derived leptin entering the CNS and regulating sympathetic tuning, including leptin penetrating into the BBB after binding to the leptin receptor (lepR), and its function as a paracrine factor, which integrates sympathetic signals through binding to adipose tissue related sensory nerve lepR 8,9. A large number of excellent reviews has discussed the molecular mechanism of the heterogeneity of the brain regulation of adipose tissues via leptin 8,9, and some related articles even summarized this kind of regulation as crosstalk between WAT and the MGBA 160. In fact, the interaction between WAT and MGBA also exists at the peripheral level, the key to which is the bidirectional regulation between leptin and intestinal microbiota. Leptin also acts on peripheral tissues, such as in skeletal muscle, kidney, pancreas, liver, heart, and gut 161. In 2011, two elegant studies found that lepR functional deficiency increased the susceptibility to *Entamoeba histolytica* significantly because of the presence of the R233 residue in the cytokine receptor homology 1 region located in the extracellular loop of the lepR, which inhibited the phosphatidylinositol-4,5-bisphosphate 3-kinase (PI3)/protein kinase B (Akt) pathway and activated caspase-3 to induce epithelial cell apoptosis 162,163. Ultimately, this not only increased susceptibility to amoeba infection, but also impaired intestinal function and destroyed the environment of the intestinal microorganisms 162-164. By contrast, the intestinal microbiota is also one of the factors necessary to maintain leptin functional homeostasis. Compared with normal mice, GF mice had more hypermethylation of CpG sites in the leptin gene promoter, but showed increased leptin secretion and leptin resistance after food intake 165. In addition, exogenous *Lactobacillus rhamnosus* GG (LGG) can improve the composition of the intestinal microbiota and reverse leptin resistance after an HFD, which is closely related to the upregulation of hypothalamic cytokine signaling-3 after LGG treatment 166 (Figure 3). Similarly, *Bacillus adolescentis* isolated from the elderly can increase the concentration of serum leptin and induce the expression of thermogenic and lipid metabolism-related genes in adipose tissue 167. Evidence also suggests that exogenous *Panax notoginseng saponins* can increase the abundance of *Akkermansia muciniphila* and *Parabacteroides distasonis* by activating leptin-AMP-activated protein kinase (AMPK)/signal transducer and activator of transcription 3 (STAT3) pathway to increase the browning potential of WAT 168. In summary, leptin is the key factor connecting WAT and the MGBA, at both the central and peripheral levels, and the latter also contains complex bidirectional regulatory relationship between intestinal microbiota and leptin. Finally, these functions enrich the metabolic potential of adipose tissue to participate in the regulation of MGBA.

### Other WAT-derived endocrine factors

Other factors secreted by WAT, including adiponectin and resistin, could interact with the MGBA the same way as leptin. Injection of testosterone cypionate or sesame oil vehicle into pregnant rats in late gestation altered the composition of the intestinal microbiota as well as promoted the biosynthesis and elongation of SCFAs. Notably, these rats also showed increased body weight, which was related to the up-regulation of *ADIPOQ* mRNA (encoding adiponectin) in inguinal WAT 169. However, the direct relationship between the changes in adiponectin level and the intestinal microbiota has not been proven. Recent studies have demonstrated that the abnormal intestinal microbiota and consequence decrease in fecal contents (especially acetate, propionate, and butyrate) of diet induced obesity (DIO) mice injected with antibiotics is associated with declining methylation of the *ADIPOQ* and *RETN* (resistin) promoters, which could be attributed to the down-regulated expression of methylation-related genes, including *DNMT1* (encoding DNA methyltransferase 1) and *DNMT3a* (encoding DNA methyltransferase 3a) 170. This experiment indirectly revealed the crosstalk among WAT-derived adiponectin, resistin, and the intestinal microbiota from an epigenetic viewpoint. Moreover, serum adiponectin and resistin levels influence cognitive function and the development of neurodegenerative diseases 171. Hence, WAT-derived adiponectin and resistin can interfere with both peripheral and central components within the MGBA.

Growing evidence underpins that there exist abundant immune cells in WAT. Endocrine factors derived from these immune cells also promote the crosstalk between WAT and other organs. The increase of circulating pro-inflammatory factors, including IL-1β, IL-6 and tumor necrosis factor (TNF)-α, impairs the BBB permeability of aged rats and attenuates tolerance to ischemic brain injury, while the removal of visceral adipose tissue decreases these inflammatory factors and partially reverses relevant brain damage 172. Such factors also function as an indirect element to foster the shifting to an aberrant CNS state. NOD-like receptor family, pyrin domain-containing 3 (NLRP3)-null mutation from visceral adipose tissue (VAT) prevents cognitive impairment in mice with Alzheimer's disease 173. Following studies reveal that NLRP3 inflammasome activates IL-1 receptor 1 in hippocampal microglia and results in cognitive impairment by releasing IL-1β 174. In addition, immune-related factors derived from WAT could also associate with intestinal homeostasis. After HFD supplemented with annatto-extracted tocotrienol treatment, the attenuation of WAT-derived IL-6 in mice could decrease the ratio of *Firmicutes/Bacteroidetes* and the abundance of *Ruminococcus lactaris*, *Dorea longicatena*, and *Lachnospiraceae* family 175. WAT-originated IL-6 can also increase the permeability of intestinal barrier, aggravating LPS-induced endotoxemia 176. Conversely, increased microbiota metabolites, such as vitamin and SCFA, down-regulate the levels of TNF-α and IL-1β in WAT 177. To sum up, there exists a complex multi-target signal pathway between WAT-derived immune factors and the MGBA. However, completely understanding these pathways is still known to be an obstacle, as functions of such immune factors are also determined by the state of body. For example, under acute psychological stress, AT-derived IL-6 promotes gluconeogenesis in the liver, which expresses a distinct function from classical pro-inflammation effects 113. Therefore, when it comes to these pathways, following studies should also pay attention to the overall state of the body.

Interestingly, WAT-derived secretory factors also include miRNAs, for example, specific metabolites of intestinal microbiota, especially tryptophan metabolites, regulate the expression of miR-181 in white adipocytes to strictly control energy expenditure, obesity, WAT inflammation, and insulin sensitivity 178. In addition, the fluctuation of central miR-181 also mediates the occurrence and development of CNS degenerative disorders and epilepsy 179,180. This metabolites-miR-181 axis constitute the framework of the interaction between WAT and the MGBA from a genetic viewpoint. However, owing to the existence of the BBB, whether WAT-derived miR-181 can regulate CNS function requires further study. In summary, the endocrine function of WAT is the core factor of its crosstalk with the MGBA. There are complex bidirectional interactions among WAT and its components as well as the whole structure of the MGBA, disorders of which constitute the pathogenesis of many metabolic diseases, providing various genetic or molecular targets to explore innovative therapeutic strategies.

### Functional heterogeneity of ADSCs and their potential to interact with the MGBA

ADSCs mainly originate from WAT, and are capable to differentiating into various tissues derived from the mesoderm, including bone, cartilage, muscle, and adipose. Their differentiation function, along with their wide availability, have meant that ADSCs have been employed as a potential tool in the field of soft tissue regeneration and reconstruction 18, with a wide application prospect to treat acute brain tissue injury and metabolic dysfunction induced by intestinal endothelial cell injury 181,182. In addition, ADSCs are involved in the regulation of the intestinal microbiota and systemic inflammation, which also demonstrates the potential of their interaction with the MGBA.

In the gut, the therapeutic roles of ADSCs are associated with increasing the diversity of the intestinal microbiota and inhibiting the abundance of pathogenic bacteria, which could reduce the secondary damage caused by intestinal mucosa injury and metabolic disorders caused by sepsis and IBD 183,184. Further studies showed that ADSCs injection accelerates intestinal epithelial cell (IEC) regeneration and activates noncanonical Wnt signal pathways to protect the intestinal mucosa against inflammatory injuries in IBD 185. Moreover, ADSCs also modulate the intestinal immune response by rebalancing the T cell repertoire 185. Generally speaking, ADSC treatment regulates the intestinal microbiota and the immune response, which interfere with the MGBA through peripheral pathways. However, the effects of ADSC administration on inflammation is also reflected in the CNS. ADSCs activate the brain-derived neurotrophic factor (BDNF)-tropomyosin related kinase B (TrkB) pathway to inhibit the inflammatory state of the brain. Similarly, ADSC treatment also upregulates nuclear factor, erythroid-2 related factor 2 (Nrf2)/heme oxygenase 1 (HO-1) signaling and decreases toll like receptor 4 (TLR4)/nuclear factor kappa B (NF-κB) activation in the CNS, which holds the M2 phenotype of macrophages, eventually alleviating the symptoms of depression 186. In addition, stereotactic transplantation of ADSCs reduces the expression of tissue edema-related AQP4, which is attributed to the inhibition of upstream p28/mitogen activated protein kinase (MAPK) and JUN N-terminal kinase (JNK) signaling in astrocytes, thus maintaining the integrity of blood-brain barrier and the functional homeostasis of the brain after intracerebral hemorrhage 187. Taken together, the interaction between ADSCs and the MGBA is not only reflected in the regulation of peripheral processes, but also in the homeostasis of brain-derived signals or pathways in the CNS.

Although modulating the components of the MGBA is emerging as a novel advantage of ADSCs, it remains difficult to verify the systematic influence of ADSCs on the whole MGBA, and there have been few studies associating ADSC-induced CNS signals with various related metabolic diseases. However, the BNDF-TrkB pathway and Nrf2 protein are not only related to inflammation in CNS, but also regulate the metabolic function of peripheral organs 188-190. BDNF and TrkB are also expressed in the gastrointestinal mucosa and are closely functionally related to the pathogenesis of obesity 191. This evidence reveals that the BDNF-TrkB pathway is significant in maintaining the metabolic regulator role of the MGBA, while its function is emerging as obviously tissue-specific in the gut and brain. Similarly, structural activation of peripheral Nrf2 signaling via intrahepatic knockout of the *Keap1* gene (encoding Kelch like ECH associated protein 1) alleviated obesity, diabetes, and liver steatosis 192,193. Hence, the BNDF-TrkB pathway, along with Nrf2 signaling, promotes the communication between the gut and brain, and are also potential research targets for ADSCs to systematically regulate the MGBA. However, it is worth noting that the research concerning same pathway in both the PNS and CNS should focus on overcoming the problem of tissue specificity (Figure 3). ADSCs is plentiful and simple to obtain, thus expanding their crosstalk with other components, including the MGBA, has a good clinical application prospect; however, the effectiveness and safety of this approach remains to be investigated.

## Conclusion and perspectives

In recent years, the previously unknown function of the MGBA has been gradually uncovered, providing numerous efficient treatment targets and innovative therapeutic modes for various diseases. From a traditional perspective, the gut and brain act as two central regulatory systems, the interaction between which contribute to connection among various kinds of diseases and ensures more systematic awareness of the pathogenesis of each disease. However, there are two obvious deficiencies in recent research. First, although studies have gradually explored the therapeutic role of the MGBA in metabolic diseases, they attached too much attention to the relationship between the microorganisms' ecosystem and metabolic diseases. In fact, the intestinal microbiota is only a peripheral initiating factor, and its subsequent induction process within the MGBA manifests as a dynamic and holistic feature. Therefore, we first reviewed the gut to brain pathway initiated by the intestinal microbiota to thoroughly analyze the pathways concerning how the MGBA dynamically regulates the development of metabolic diseases from regional to holistic perspectives. Second, brain-derived signals, together with the MGBA, also promote the incidence of various metabolic diseases indirectly, especially obesity, mainly through the HPA axis or the ANS, which constitutes the brain to gut pathway of the MGBA. These two pathways form a bidirectional regulatory framework within the MGBA, determining the molecular basis of which will lead to a deeper understanding of the dynamic processes of metabolic diseases induced by the MGBA. Most metabolic diseases belong to the category of chronic diseases; therefore, future studies should focus on exploring the dynamic molecular changes within the MGBA during the progress of metabolic diseases to optimize therapeutic strategies at different stages of the disease.

Generally, research achievements based on the MGBA have not been translated satisfactorily to clinical applications in metabolic diseases. Therefore, the latter part of this paper mainly explored the interaction between peripheral organs, such as different kinds of AT, and the MGBA, to enrich the theoretical basis of the MGBA in the treatment of metabolic diseases. There is heterogeneity in the crosstalk between different kinds of AT and the MGBA; therefore, the molecular basis of their differences should be determined. In particular, analysis of ADSCs showed that their interaction with the MGBA has promising implications for the innovation of therapeutic strategies concerning metabolic diseases. It is worth noting that the abundant nerve fibers and efficient endocrine function of AT provide anatomical and physiological foundations for its interplay with the MGBA, suggesting that this interaction involves the integration of neuroimmunology and endocrine metabolism. In conclusion, future research should continue to screen the key molecular targets or pathways for the treatment of metabolic diseases, and pay attention to the dynamics of the different stages of these diseases and the significance of multidisciplinary integration to study the development of metabolic diseases.

## Abbreviations

MGBA: microbiota-gut-brain axis
AT: adipose tissue
WAT: white adipose tissue
ADSCs: adipose tissue derived mesenchymal stem cells
T2DM: type 2 diabetes mellitus
CNS: central nervous system
BBB: blood brain barrier
ANS: autonomic nervous system
miRNAs: microRNAs
PYY: peptide YY
CCK: cholecystokinin
GLP-1: glucagon like peptide-1
FFA2: free fatty acid receptor 2
RYGB: Roux-en-Y gastric bypass
GF: germ-free
5-HT: serotonin
GABA: gamma-aminobutyric acid
ssRNA: single-stranded RNA
FMT: fecal microbiota transplantation
VAN: vagal afferent neuron
ENS: enteric nervous system
ARC: arcuate nucleus
PVN: paraventricular nucleus
AP: area postrema
BAT: brown adipose tissue
SCFAs: short-chain fatty acids
FGIDs: functional gastrointestinal diseases
IBS: irritable bowel syndrome
HPA: hypothalamic-pituitary-adrenal axis
MDD: major depressive disorder
MCFAs: medium-chain fatty acids
Treg: regulatory T cell
Th17: T helper cell 17
Teff17: effector Th 17
EAE: encephalomyelitis
FIP: frame-projected independent-fiber photometry
SNS: sympathetic nervous system
NRG4: neuregulin 4
BMP8B: bone morphogenetic protein 8b
IL: interleukin
VMN: ventromedial hypothalamic nucleus
CCKA: cholecystokinin A
ApoA-IV: apolipoprotein A-IV
DIT: diet-induced thermogenesis
FGF19: fibroblast growth factor 19
MSNA: muscle sympathetic nervous activity
TRPV1: transient receptor potential vanilloid 1
POMC: proopiomelanocortin
UCP1: uncoupling protein 1
HFD: high fat diet
EODF: every-other-day fasting
IF: intermittent fasting
Fiaf: fasting-induced adipocyte factor
FAT: fatty acid translocase
PA: palmitic acid
lepR: leptin receptor
LGG: Lactobacillus rhamnosus GG
AMPK: leptin-AMP-activated protein kinase
STAT3: signal transducer and activator of transcription 3
DIO: diet induced obesity
TNF: tumor necrosis factor
NLRP3: NOD-like receptor family: pyrin domain-containing 3
VAT: visceral adipose tissue
IEC: intestinal epithelial cell
BDNF: brain-derived neurotrophic factor
TrkB: tropomyosin related kinase B
Nrf2: erythroid-2 related factor 2
HO-1: heme oxygenase 1
TLR4: toll like receptor 4
NF-B: nuclear factor kappa B
MAPK: mitogen activated protein kinase
JNK: JUN N-terminal kinase
